# Lycopene Attenuates Acute Cyclophosphamide-Induced Renal Injury in Mice: Renal Function, Cytokine Transcription, and Histopathological Evidence

**DOI:** 10.3390/ph19091415

**Published:** 2026-09-07

**Authors:** Elif Ece Akgun, Esra Bilici, Busra Gulbenli Turkoglu

**Affiliations:** 1 Department of Histology and Embryology, Faculty of Veterinary Medicine, Ataturk University, 25240 Erzurum, Türkiye; 2 Laborant and Veterinary Health Program, Eşme Vocational School, Uşak University, 64600 Uşak, Türkiye; 3 Department of Pathology, Faculty of Veterinary Medicine, Mehmet Akif Ersoy University, 15000 Burdur, Türkiye; bgulbenliturkoglu@mehmetakif.edu.tr

**Keywords:** cyclophosphamide, lycopene, nephrotoxicity, pro-inflammatory cytokines, renal histopathology, oxidative stress, murine model

## Abstract

**Background/Objectives:** Cyclophosphamide (CP) can produce acute renal toxicity through overlapping oxidative, inflammatory, and apoptotic processes. Although lycopene is widely studied for antioxidant and anti-inflammatory activity, its renal effects during acute CP exposure remain incompletely defined. We investigated whether a 10-day course of oral lycopene before CP administration altered early biochemical, transcriptional, histological, and apoptosis-related renal responses. **Methods:** Twenty-eight male CD-1 mice were assigned equally to control, lycopene (20 mg kg^−1^ day^−1^), CP (200 mg kg^−1^ intraperitoneally on day 10), or CP + lycopene groups (n = 7). At 24 h after CP administration, serum BUN and creatinine were measured together with renal TBARS-derived MDA-equivalent values, SOD activity, TNF-α/IL-6/IL-1β mRNA abundance, histopathological injury, and cleaved caspase-3 immunoreactivity. **Results:** CP increased BUN, creatinine, and renal TBARS-derived MDA-equivalent values; decreased SOD activity; increased all three inflammatory transcripts; worsened tubular and tubulointerstitial injury; and increased cleaved caspase-3 labeling. Compared with CP alone, lycopene pretreatment reduced BUN, renal TNF-α, IL-6, and IL-1β mRNA, tubular and tubulointerstitial damage, and cleaved caspase-3 labeling. Creatinine, renal TBARS-derived MDA-equivalent values, and SOD activity were not significantly different between the CP and CP + lycopene groups. **Conclusions:** In this prophylactic model (20 mg kg^−1^ day^−1^ for 10 days; 24 h assessment), lycopene provided partial early protection against CP-associated renal injury. The response was evident in BUN, inflammatory transcription, tissue injury, and apoptotic labeling, but not in creatinine or the two measured redox indices. The study does not establish pathway causality and does not support extrapolation to post-injury treatment or long-term protection.

## 1. Introduction

Cyclophosphamide (CP) is an oxazaphosphorine prodrug used in oncology and in selected immune-mediated diseases. Its usefulness is counterbalanced by dose-dependent injury to non-target tissues, including the kidney. Hepatic activation generates phosphoramide mustard, responsible for DNA alkylation, and acrolein, a highly reactive aldehyde implicated in urothelial and renal toxicity. Oxidant formation and lipid peroxidation after CP exposure can disrupt tubular-cell membranes and promote secondary inflammatory signaling [1,2].

CP-induced nephrotoxicity reflects several interacting processes rather than a single dominant pathway [3]. Experimental studies frequently report increased lipid peroxidation together with depressed antioxidant-enzyme activity, including lower SOD and catalase, indicating disruption of renal redox balance [4]. Evidence from plant-derived interventions also supports oxidative injury as an important, although not exclusive, component of CP toxicity [5,6,7]. Oxidative perturbation may in turn enhance NF-κB-responsive inflammatory transcription, including TNF-α, IL-1β, and IL-6. At the tissue level, CP injury is commonly expressed as tubular epithelial vacuolation or dilatation, vascular congestion, and inflammatory-cell accumulation, with concurrent increases in circulating BUN and creatinine [8,9,10].

Because these processes occur simultaneously, candidate protective agents should ideally be evaluated across functional, biochemical, inflammatory, and structural outcomes rather than through a single marker. Dietary phytochemicals are of interest because individual compounds can influence several injury pathways at experimentally tolerable doses [11,12]. Nevertheless, organ-specific pharmacokinetics and the nature of the toxic insult can materially alter the response; evidence from hepatic or cardiac injury therefore cannot be assumed to apply directly to the kidney [10].

Lycopene is an acyclic C_40_ carotenoid abundant in tomatoes and tomato products [13], with strong singlet-oxygen-quenching capacity [14]. Experimental work also indicates effects on lipid peroxidation and endogenous antioxidant defenses [15,16], as well as modulation of inflammatory signaling, including NF-κB-related responses [17]. Renal protection has been described in several non-CP settings, including ischemia–reperfusion, cisplatin exposure, endotoxin-associated injury, and drug-related nephrotoxicity [9,10,18]. CP differs from many of these insults because its reactive metabolite, acrolein, can rapidly disrupt thiol/redox homeostasis and initiate inflammatory injury. Accordingly, the renal response to lycopene during acute CP exposure should be judged across function, redox biochemistry, inflammatory transcription, histology, and apoptosis-related readouts rather than inferred from lycopene’s established antioxidant reputation.

The present experiment therefore addressed a focused prophylactic question: whether oral lycopene at 20 mg kg^−1^ day^−1^ for 10 days modifies the early renal response to a single 200 mg kg^−1^ CP challenge in male CD-1 mice. The prespecified readouts were serum BUN and creatinine, renal TBARS-derived MDA-equivalent values and SOD activity, renal TNF-α, IL-6, and IL-1β transcripts, semiquantitative histopathology, and cleaved caspase-3 immunoreactivity. We anticipated that lycopene pretreatment would attenuate CP-associated abnormalities across these endpoints.

## 2. Results

### 2.1. Renal Function Markers

**Figure 1:**
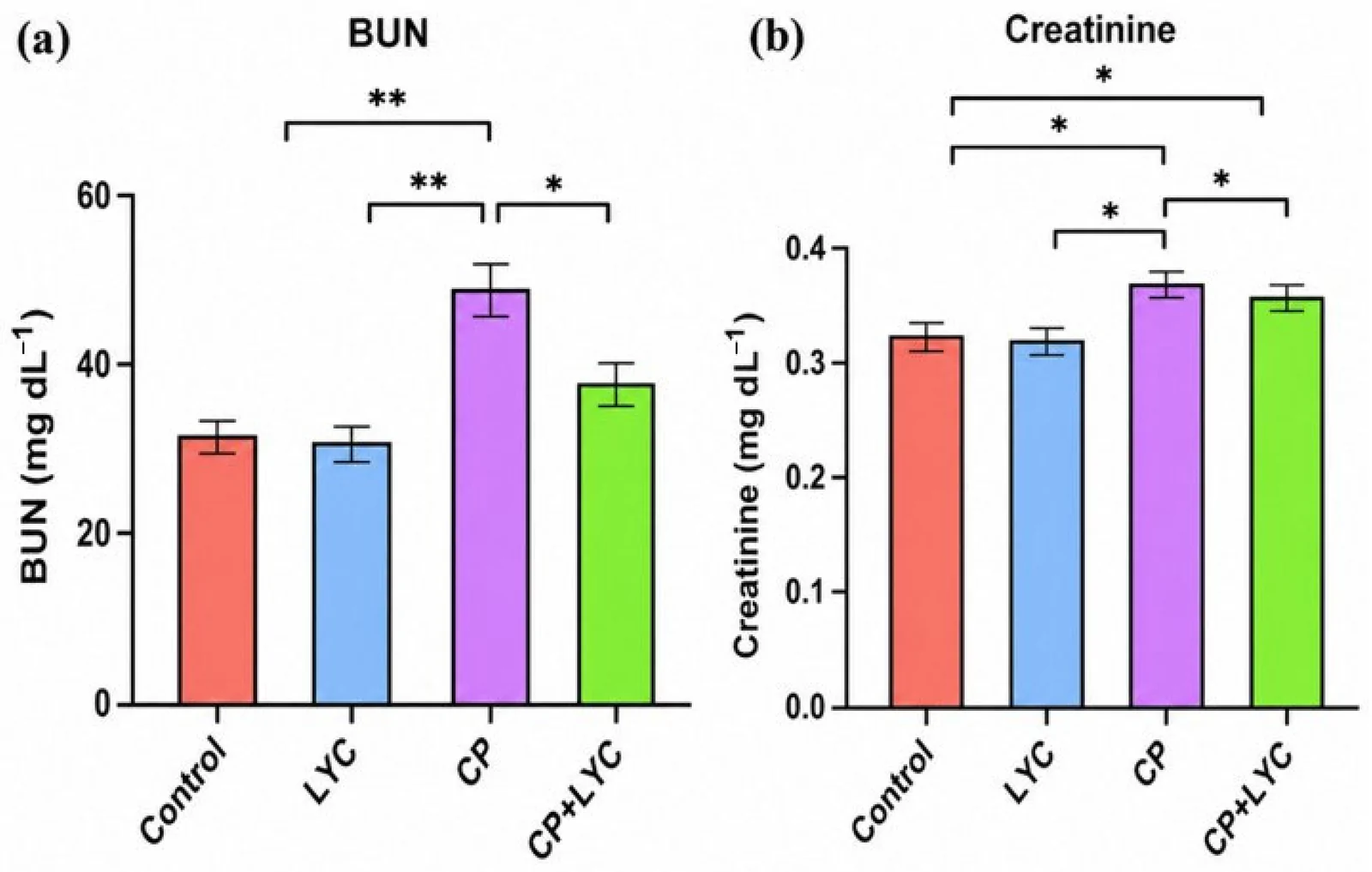
Serum indices of renal function. Panel (**a**) presents blood urea nitrogen (BUN) and panel (**b**) serum creatinine for control, LYC, CP, and CP + LYC mice. Data are shown as mean ± SEM for seven animals per group. Overall group effects were tested by one-way ANOVA, followed by Tukey HSD comparisons. Brackets mark significant differences (* *p* < 0.05; ** *p* < 0.01).

Both serum BUN and creatinine increased after CP administration relative to control (*p* < 0.01 and *p* < 0.05, respectively; Figure 1a,b), whereas neither marker changed in mice receiving lycopene alone. Within the CP-exposed animals, pretreatment with lycopene reduced BUN (*p* < 0.05 versus CP), but creatinine remained statistically indistinguishable from CP alone and higher than control (*p* < 0.05). Thus, at 24 h, improvement in the measured renal biochemical indices was limited to BUN.

### 2.2. Oxidative Stress Parameters

**Figure 2:**
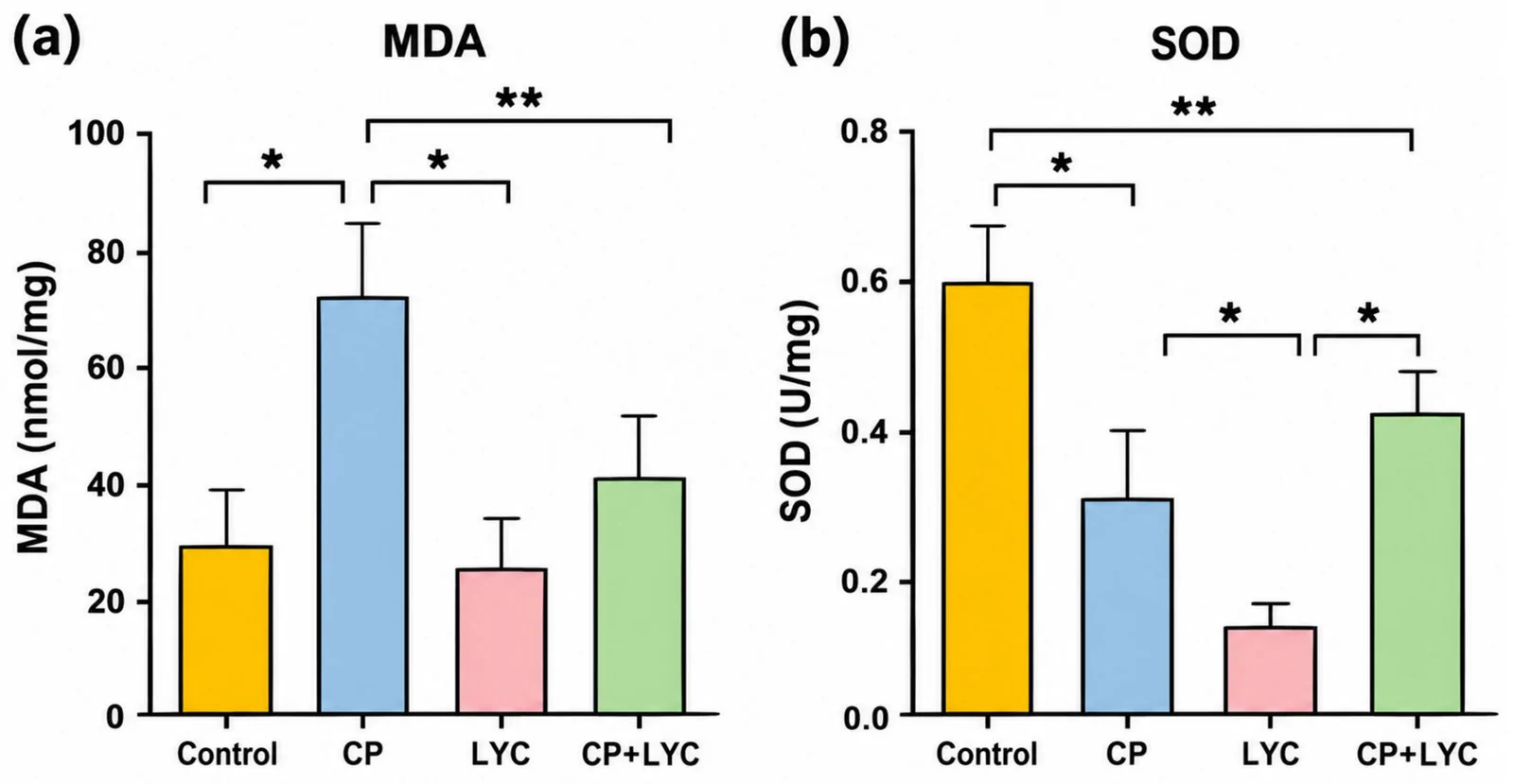
Renal redox measurements. Thiobarbituric acid-reactive substances (TBARS), reported as MDA equivalents (**a**), and superoxide dismutase (SOD) (**b**) activity was quantified in control, LYC, CP, and CP + LYC kidneys. Bars represent mean ± SEM (n = 7). Following one-way ANOVA, Tukey HSD was applied when the omnibus test was significant. Brackets identify pairwise differences (* *p* < 0.05; ** *p* < 0.01).

Relative to control, CP increased the renal TBARS-derived MDA-equivalent signal and decreased SOD activity (both *p* < 0.05; Figure 2a,b). The LYC-only group did not differ significantly from control for either marker. TBARS-derived MDA-equivalent values and SOD activity in CP + LYC mice were not significantly different from CP alone (both *p* > 0.05). Accordingly, this experiment detected no lycopene-related improvement in these two renal redox measures at 24 h.

### 2.3. Pro-Inflammatory Cytokine mRNA Expression

**Figure 3:**
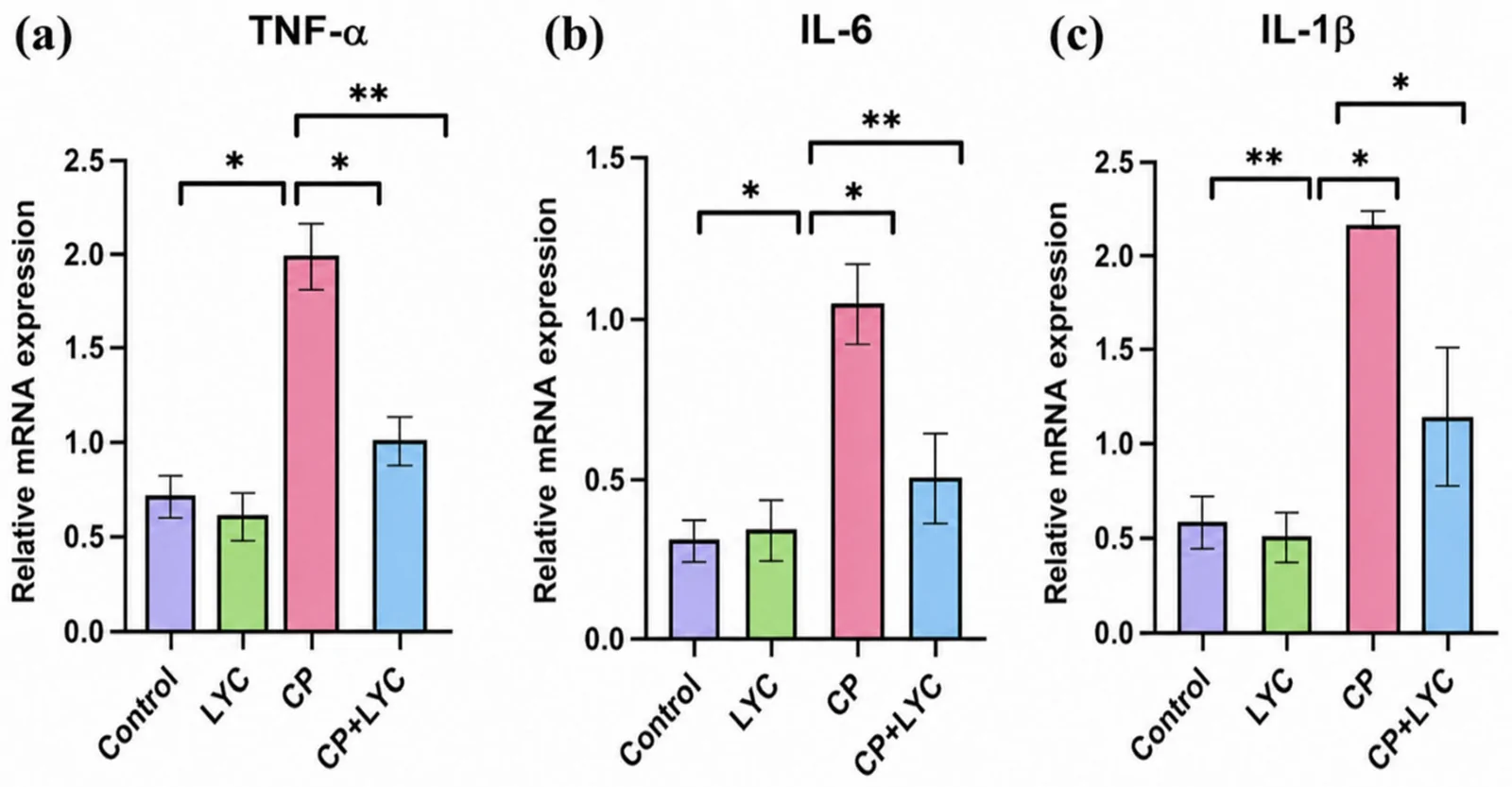
Renal expression of pro-inflammatory cytokine transcripts. Relative TNF-α (**a**), IL-6 (**b**), and IL-1β (**c**) mRNA levels are shown for control, LYC, CP, and CP + LYC mice. Expression was referenced to β-actin and calculated by the 2^−ΔΔCt^ method. Values are mean ± SEM (n = 7). One-way ANOVA followed by Tukey HSD was used for inferential testing; brackets indicate significant differences (* *p* < 0.05; ** *p* < 0.01).

Renal inflammatory transcripts responded strongly to CP: TNF-α, IL-6, and IL-1β mRNA were each higher than control (all *p* < 0.05; Figure 3a–c). Lycopene alone had no detectable effect on these transcripts (all *p* > 0.05). In contrast, each transcript was significantly lower in the CP + LYC group than in mice given CP alone (all *p* < 0.05), demonstrating attenuation of the CP-associated transcriptional inflammatory response.

### 2.4. Histopathological Findings

**Figure 4:**
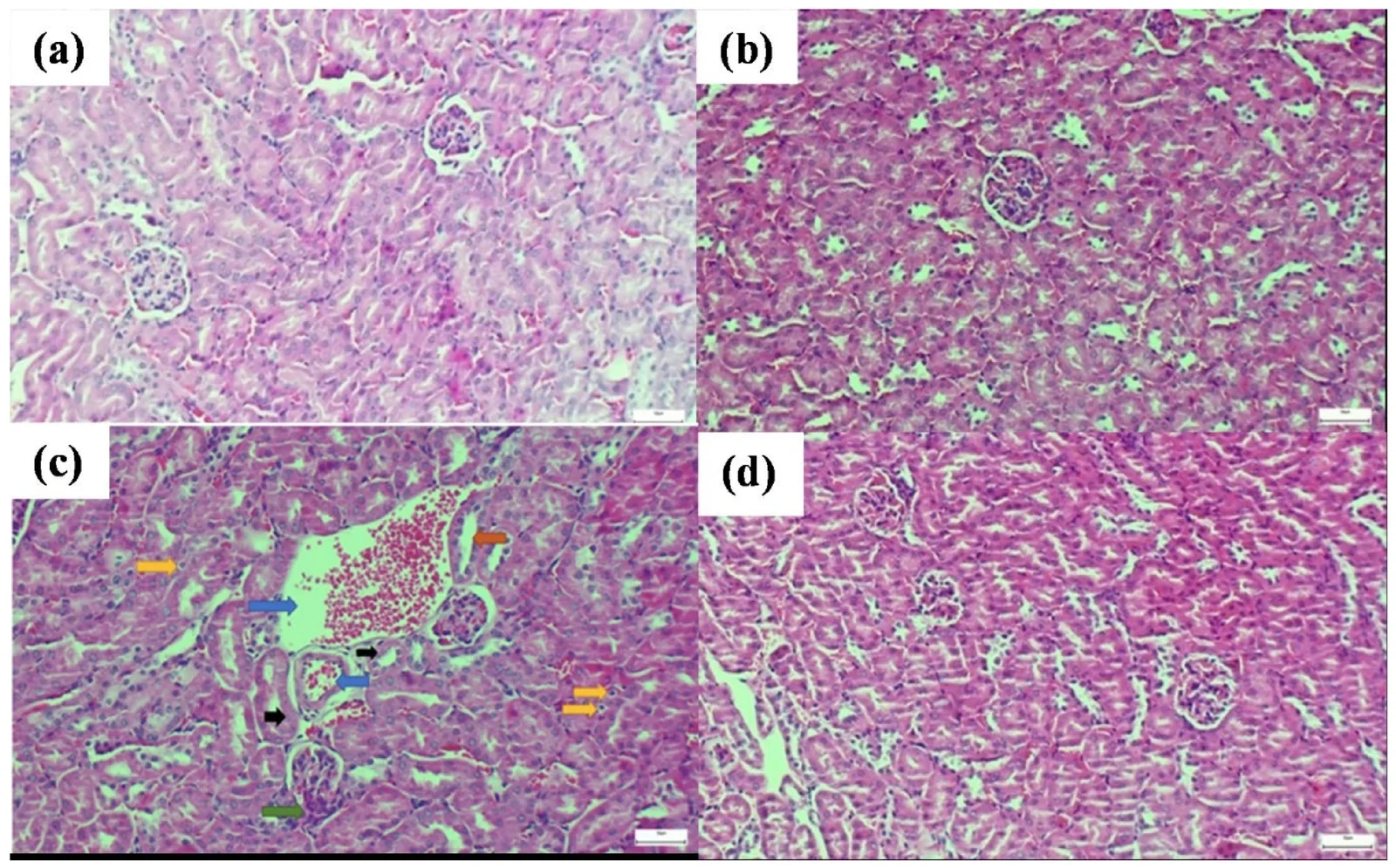
Renal histopathology in representative H&E-stained sections. Images correspond to (**a**) control, (**b**) LYC, (**c**) CP, and (**d**) CP + LYC mice. Control and LYC kidneys show preserved architecture. In CP-treated kidneys, arrows identify vascular congestion (blue), tubular epithelial vacuolization (yellow), tubular dilatation (orange), epithelial desquamation (black), and leukocyte infiltration (green). These lesions are visibly less extensive, although still present, in the CP + LYC group. Scale bar = 50 µm.

Renal architecture was preserved in control and LYC mice (Figure 4a,b). CP produced prominent tubular epithelial vacuolization and dilatation, vascular congestion, and interstitial leukocyte infiltration (Figure 4c). These abnormalities were less extensive after lycopene pretreatment, although residual injury remained evident (Figure 4d). The composite renal damage score was higher with CP than with control or LYC (*p* < 0.05) and lower in CP + LYC than in CP alone (*p* < 0.05), while remaining above the control (*p* < 0.05). Subscore analysis showed lower tubular injury (1.29 ± 0.15 vs. 2.43 ± 0.18, *p* < 0.05) and tubulointerstitial injury (1.14 ± 0.12 vs. 2.14 ± 0.14, *p* < 0.05) in CP + LYC versus CP. The difference in glomerular score was not significant (0.57 ± 0.08 vs. 0.86 ± 0.10, *p* > 0.05).

### 2.5. Cleaved Caspase-3 Immunoreactivity

**Figure 5:**
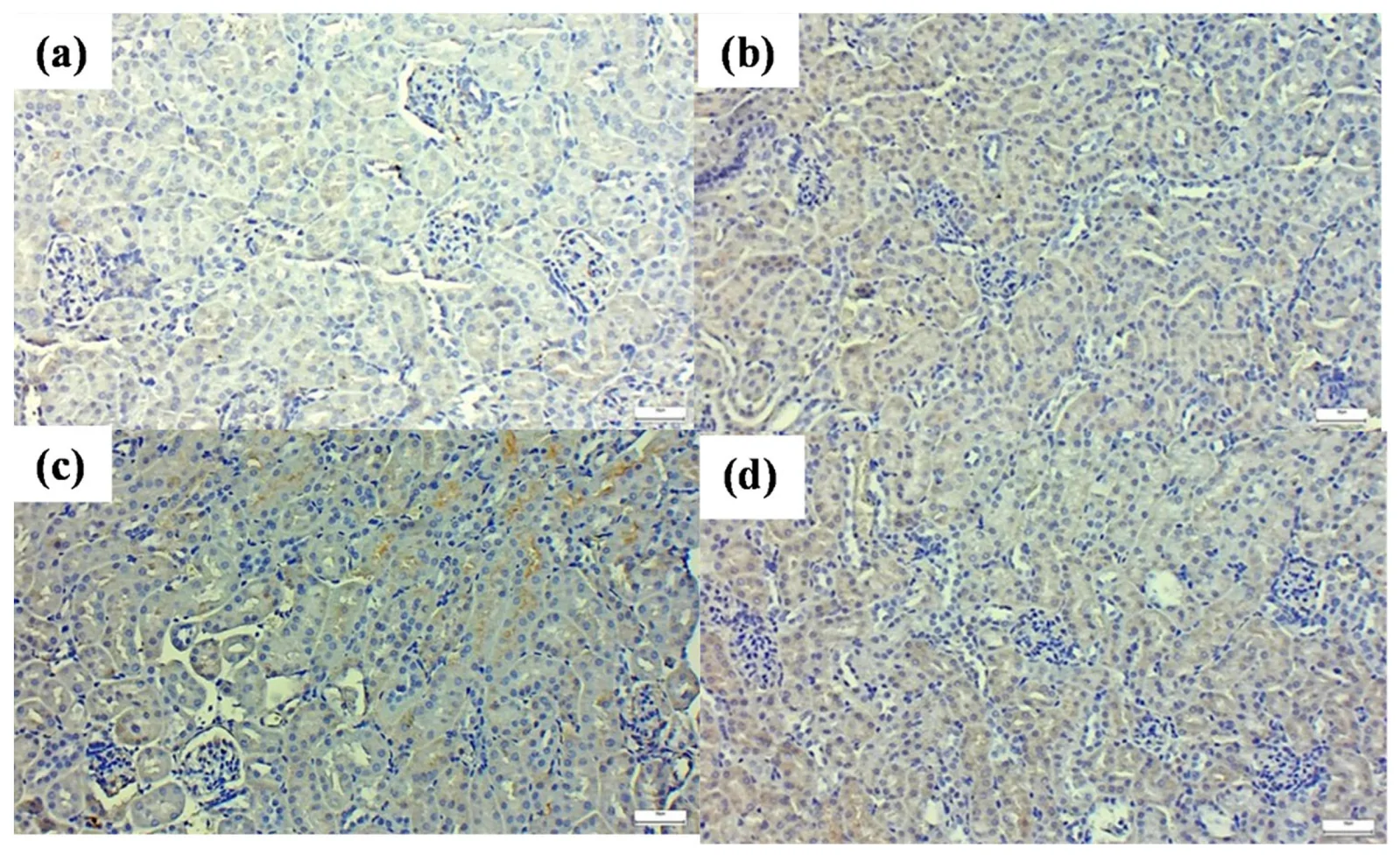
Representative cleaved caspase-3 immunostaining in mouse kidney. Sections from (**a**) control, (**b**) LYC, (**c**) CP, and (**d**) CP + LYC groups are shown. Brown cytoplasmic staining in tubular epithelial cells was sparse in control and LYC kidneys, pronounced after CP, and less extensive after lycopene pretreatment. Sections were counterstained with Mayer’s hematoxylin. Scale bar = 50 µm.

Cleaved caspase-3 staining was sparse in control and LYC kidneys but prominent in tubular epithelial cells after CP exposure (Figure 5a–c). Staining was reduced, but not normalized, in CP + LYC mice (Figure 5d). The labeling index was 18.74 ± 1.65% in the CP group versus 2.41 ± 0.38% in control and 2.18 ± 0.32% in LYC (*p* < 0.01). Lycopene pretreatment reduced the index to 9.82 ± 1.12% compared with CP alone (*p* < 0.05), although it remained above control (*p* < 0.05).

## 3. Discussion

Overall, lycopene pretreatment produced a selective rather than uniform protective response to acute CP nephrotoxicity. The clearest improvements were lower BUN, reduced renal TNF-α, IL-6, and IL-1β transcription, lower tubular and tubulointerstitial injury scores, and less cleaved caspase-3 immunoreactivity. By contrast, creatinine did not significantly decline, and renal TBARS-derived MDA-equivalent values and SOD activity did not differ from CP alone. The data should therefore be interpreted as partial early protection, not as complete restoration of renal function or confirmation of a generalized antioxidant action. Within the 24 h observation window, the strongest signal was seen in inflammatory transcription, tissue injury, and apoptosis-related labeling. Because we did not measure cytokine proteins, upstream NF-κB activation, or a broader redox profile, these relationships remain associative.

BUN and creatinine require separate interpretation. BUN is a commonly used biochemical marker in the assessment of renal function, but it is not a direct measure of glomerular filtration and can vary with extra-renal determinants, including hepatic urea production, dietary protein/protein catabolism, and hydration status; serum creatinine is likewise an indirect filtration surrogate with its own kinetic limitations [19,20,21]. In the present experiment, BUN was lower with lycopene pretreatment, whereas creatinine was unchanged relative to CP alone. This pattern is compatible with partial biochemical protection rather than complete restoration of renal excretory function [18,22]. Other experimental lycopene studies have reported concurrent reductions in BUN and creatinine after ischemia–reperfusion or lipopolysaccharide-associated renal injury [10,18,23]. Differences in insult, exposure schedule, lycopene dose, and sampling time could explain the discrepancy. Importantly, a single 24 h measurement cannot determine whether the creatinine response would subsequently recover, persist, or worsen; longitudinal sampling is required.

The redox results were likewise specific to the endpoints measured. CP increased the TBARS-derived MDA-equivalent signal and decreased SOD activity, changes compatible with acute oxidative injury, yet lycopene did not significantly alter either measure relative to CP alone. Pektaş et al. [24] reported similarly modest short-term effects in renal tissue after ischemia–reperfusion, whereas Kaya et al. [25] found that serum and tissue MDA did not respond identically. Other models have shown lower MDA together with greater SOD activity, including streptozotocin-associated nephropathy [26] and diclofenac-related acute kidney injury [27]. Such variability is biologically plausible because matrix, injury mechanism, dose duration, and sampling time all influence redox readouts. Because the TBARS assay is not chemically specific for MDA and can detect other thiobarbituric acid-reactive compounds, the signal should be interpreted as an MDA-equivalent index of lipid-peroxidation-related reactivity rather than as a direct measurement of tissue MDA. Because this experiment assessed only the TBARS-derived signal and total SOD activity, it cannot address possible effects on catalase, glutathione-dependent defenses, total antioxidant capacity, reactive nitrogen species, or other components of redox regulation.

The most consistent molecular response was reduced renal TNF-α, IL-6, and IL-1β mRNA after lycopene pretreatment. Previous studies have linked lycopene to modulation of NF-κB-related inflammatory signaling, and the lower leukocyte infiltration observed histologically is directionally compatible with the transcript findings [22,23]. Nevertheless, this study measured cytokine mRNA rather than cytokine protein and did not quantify NF-κB activation. The appropriate interpretation is therefore suppression of inflammatory gene expression associated with tissue protection, rather than demonstration of a specific signaling mechanism.

Cleaved caspase-3 labeling was also lower in CP + LYC than in CP alone, supporting reduced execution-phase apoptotic activity. The literature links inflammatory and redox perturbations to caspase-dependent cell death in renal injury [28,29]. Our design, however, cannot determine which upstream pathway produced this change. Bax/Bcl-2 balance, cytochrome c release, initiator caspases, and pathway inhibition were not assessed. Consequently, the parallel reductions in cytokine transcripts, cleaved caspase-3, and tissue injury should be regarded as concordant observations rather than a causal sequence.

Several limitations define the scope of inference. We studied only one lycopene dose and a prophylactic 10-day regimen, so dose–response relationships and treatment after established renal injury remain unknown. Sampling was restricted to 24 h after CP, preventing assessment of delayed injury, repair, or response robustness. The 200 mg kg^−1^ CP challenge was strictly a single-dose preclinical model and should not be interpreted as dose-equivalent to routine fractionated human chemotherapy. Healthy non-tumor-bearing male mice were used; therefore, sex-dependent responses and any effect of lycopene on CP antineoplastic efficacy were not evaluated. The redox assessment was limited to a TBARS-derived MDA-equivalent signal and total SOD activity; cytokines were measured at the mRNA level only, and we did not perform pathway-specific experiments. Future work should include multiple doses and time points, therapeutic or post-CP arms, tumor-bearing models, broader redox profiling, cytokine protein measurements, and direct interrogation of NF-κB and apoptosis pathways.

Within these constraints, lycopene pretreatment produced a coherent reduction in several early CP-associated renal injury readouts, but the response was incomplete. The present study therefore supports further mechanistic and longitudinal testing rather than immediate clinical extrapolation.

## 4. Materials and Methods

### 4.1. Drugs and Chemicals

Cyclophosphamide (Endoxan^®^, Baxter Oncology GmbH, Halle (Westfale) Germany) was purchased through a local pharmacy and prepared immediately before administration in sterile 0.9% sodium chloride. Lycopene (Redivivo^®^, DSM Nutritional Products France, Village-Neuf, France) was used as the test compound.

### 4.2. Animals

Ethical approval was granted by the Burdur Mehmet Akif Ersoy University Animal Ethics Committee (approval 1445, 12 March 2025). The experiment used 28 male CD-1 mice, 6–8 weeks of age and weighing 20–30 g, obtained from the university Experimental Animals Center, Faculty of Veterinary Medicine. Mice were kept in polypropylene cages at 23 ± 1 °C under a 12:12 h light–dark schedule and had free access to standard feed and water.

### 4.3. Experimental Design

A computer-generated allocation sequence was used to assign seven mice to each of four experimental groups. Oral administrations were given by gavage once daily on days 1–10. Lycopene was prepared in corn oil, and the same volume of corn-oil vehicle was administered to animals not receiving lycopene. On day 10, the CP groups received freshly prepared cyclophosphamide intraperitoneally in 0.9% sodium chloride; the corresponding non-CP groups received intraperitoneal saline.

**Figure 6:**
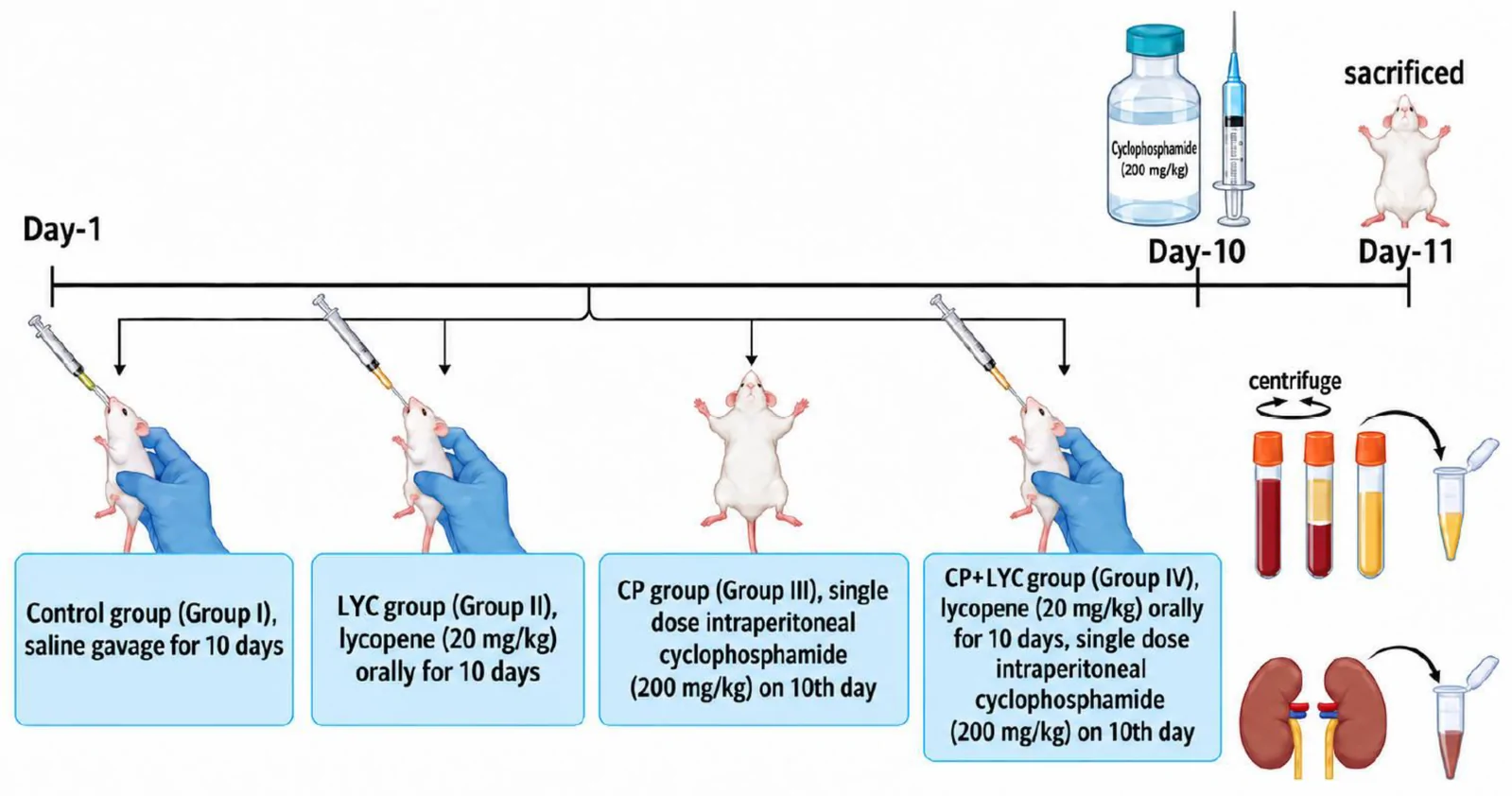
Schematic timeline of the experimental design.

Treatments were as follows: control, corn oil for 10 days plus intraperitoneal saline on day 10; LYC, lycopene 20 mg kg^−1^ day^−1^ for 10 days plus intraperitoneal saline on day 10; CP, corn oil for 10 days plus CP 200 mg kg^−1^ intraperitoneally on day 10; and CP + LYC, lycopene 20 mg kg^−1^ day^−1^ for 10 days plus CP 200 mg kg^−1^ intraperitoneally on day 10. Figure 6 summarizes the schedule. The lycopene dose was selected from prior preclinical nephroprotection work. No deaths occurred during pretreatment or the subsequent 24 h observation period. CP-exposed mice showed transient lethargy and approximately 3–5% acute body-weight loss; the apparent attenuation of weight loss in CP + LYC mice was descriptive and was not treated as a primary efficacy outcome.

### 4.4. Sample Collection

At 24 h after the day-10 treatment, mice were weighed and anesthetized intraperitoneally with freshly prepared ketamine (87.5 mg kg^−1^) plus xylazine (12.5 mg kg^−1^) [30]. Animals were maintained on a warmed platform. We confirmed adequate terminal anesthesia by loss of righting, pedal withdrawal, and palpebral reflexes before blood collection; we monitored respiration and body coloration continuously.

While animals remained deeply anesthetized, blood was collected slowly by cardiac puncture with a 23–25-gauge needle. After exsanguination, cervical dislocation was performed as a secondary euthanasia method. Absence of respiration and cardiac activity was confirmed before tissue collection.

Blood was allowed to clot and was centrifuged at 3000× *g* for 10 min; serum was transferred to sterile microcentrifuge tubes. Kidneys were excised promptly, rinsed in cold sterile saline, and blotted dry. Tissue designated for histology and immunohistochemistry was fixed in 10% neutral-buffered formalin, whereas remaining renal tissue was rapidly frozen and stored at −80 °C until biochemical analysis or RNA extraction.

### 4.5. Serum Biochemical and Oxidative Stress Analyses

Serum BUN and creatinine were quantified with commercial reagents from DiaSys Diagnostic Systems GmbH (Holzheim, Germany; BUN, Cat. No. 1 3101 99 10 021; Creatinine FS, Cat. No. 1 1711 99 10 021) and expressed in mg dL^−1^. Renal TBARS reactivity, reported as MDA equivalents, and total SOD activity were measured in kidney homogenates with Cayman Chemical assays (Ann Arbor, MI, USA; TBARS, Item No. 10009055; Superoxide Dismutase Assay, Item No. 706002).

For the TBARS determination, 25 mg of kidney was homogenized on ice with 250 µL of chilled radioimmunoprecipitation buffer supplemented with protease inhibitor. The homogenate was centrifuged for 10 min at 1600× *g* and 4 °C, and the supernatant was used in the TBARS procedure. Sodium dodecyl sulfate and thiobarbituric acid reagent were then added. After heating at 95 °C for 60 min, tubes were cooled on ice for 5 min and centrifuged again at 1600× *g* for 10 min. Absorbance was recorded at 532 nm. Because the TBARS reaction is not chemically specific for MDA, we converted the signal using the MDA standard curve and reported TBARS values as MDA equivalents rather than as a direct measure of tissue MDA concentration.

For total SOD activity, renal tissue was homogenized at a 1:10 tissue-to-buffer ratio in ice-cold 20 mM HEPES (pH 7.2) containing 1 mM EGTA, 210 mM mannitol, and 70 mM sucrose. After centrifugation at 1500× *g* for 5 min at 4 °C, the supernatant was retained. Samples were diluted as required to remain within the assay range, incubated with the radical detector and xanthine oxidase for 30 min at room temperature, and read at 450 nm.

The protein content of each renal homogenate was quantified with the BCA Protein Assay Kit (Thermo Fisher Scientific, Rockford, IL, USA; Cat. No. 23225). TBARS-derived MDA-equivalent and SOD values were normalized to protein content and expressed as nmol MDA equivalents mg^−1^ protein and U mg^−1^ protein, respectively. Every biochemical sample was assayed in duplicate.

### 4.6. Quantitative Real-Time RT-PCR

Frozen kidney samples were used for RNA isolation with the A.B.T.™ Blood/Tissue RNA Purification Kit (Atlas Biotechnology Laboratory Materials Industry and Trade Ltd., Ankara, Türkiye; Cat. No. I04-01-10), following the supplier’s procedure [31]. Use of this extraction approach for kidney material has also been reported previously [32]. RNA quantity and purity were checked spectrophotometrically; A_260_/A_280_ values ranged from 1.85 to 2.05. Integrity was verified by microfluidic electrophoresis, with distinct 28S/18S rRNA profiles and RIN values ≥ 8.0 in all samples before reverse transcription.

One microgram of total RNA was reverse-transcribed with the PrimeScript™ RT Reagent Kit (Perfect Real Time; Takara Bio Inc., Kusatsu, Shiga, Japan; Cat. No. RR037A) following the manufacturer’s protocol.

Real-time PCR amplification was carried out with an Applied Biosystems 7300 instrument using SYBR^®^ Green PCR Master Mix (Thermo Fisher Scientific, Waltham, MA, USA; Cat. No. 4344463) and the primer sets listed in Table 1. Reactions began with 10 min at 95 °C and were followed by 40 cycles comprising 15 s at 95 °C, 60 s at 60 °C, and 20 s at 72 °C. Three technical reactions were run for every biological sample.

The mean cycle-threshold (Ct) value from the three technical replicates was used for each biological sample. Target-gene expression was normalized to β-actin and expressed relative to the control group with the 2^−ΔΔCt^ method [33]. Raw β-actin Ct values were stable across groups (control, 18.42 ± 0.18; LYC, 18.35 ± 0.15; CP, 18.51 ± 0.21; CP + LYC, 18.46 ± 0.19; one-way ANOVA, *p* > 0.05). Standard curves generated from serial 10-fold dilutions yielded amplification efficiencies of 94.8–102.3% and R^2^ > 0.99 for all target and reference primer sets.

### 4.7. Histopathological Analysis

Kidneys fixed in neutral-buffered formalin were processed through graded ethanol and xylene before paraffin embedding. Sections were cut at 4 µm and stained with hematoxylin and eosin. Histological examination was performed with a Zeiss Axioscope 5 light microscope (Carl Zeiss Microscopy GmbH, Oberkochen, Germany).

An experienced histologist who was blinded to group allocation scored all sections. We recorded a randomly selected 20% subset in a blinded manner, yielding >90% intra-observer concordance. For each animal, we evaluated 20 non-overlapping cortical fields at 200× magnification. Tubular injury criteria comprised degeneration or vacuolization, brush-border loss, dilatation, desquamation, necrosis, and intraluminal casts. Glomerular assessment considered structural changes and Bowman’s space, whereas the tubulointerstitial domain included inflammatory-cell infiltration, hemorrhage, and vascular congestion.

Each histological domain was graded from 0 to 3 according to the proportion of the assessed area affected: 0, no lesion; 1, <25%; 2, 26–50%; and 3, >50%. We averaged domain scores for each animal, which served as the experimental unit. We adapted the scoring framework from published semiquantitative renal pathology methods [31].

### 4.8. Immunohistochemical Analysis of Cleaved Caspase-3

For cleaved caspase-3 immunohistochemistry, 4 µm paraffin sections were placed on Superfrost Plus slides. Sections underwent two 15 min xylene steps, followed by graded rehydration. Antigen retrieval was achieved by microwave heating in citrate buffer (pH 6.0). After cooling, endogenous peroxidase was quenched for 5 min with 3% hydrogen peroxide, and nonspecific sites were blocked for 20 min at room temperature with 2.5% normal horse serum.

The primary antibody, rabbit polyclonal anti-cleaved caspase-3 (Asp175; Cell Signaling Technology, Danvers, MA, USA; Cat. No. 9661), was applied at 1:400 and incubated overnight at 4 °C. After washing with phosphate-buffered saline, immunoreactivity was visualized with the ImmPRESS^®^ Excel Amplified Polymer Anti-Rabbit IgG Peroxidase system (Vector Laboratories, Newark, CA, USA; Cat. No. MP-7601) and ImmPACT^®^ DAB. Mayer’s hematoxylin was used as the counterstain before dehydration, clearing, and mounting in Entellan.

A group-blinded observer quantified immunostaining in 10 randomly selected, non-overlapping renal-cortical fields per animal at 400× magnification using a Zeiss Axioscope 5 microscope. Tubular epithelial cells with distinct brown diaminobenzidine staining were counted as cleaved caspase-3 positive. We enumerated positive and total tubular epithelial cells to calculate the labeling index.

$$Cleaved\;caspase-3\;labeling\;index\left(\%\right)=\frac{Number\;of\;positive\;cells}{Total\;number\;of\;cells\;counted}\times 100$$

We calculated the mean labeling index across the 10 fields for each animal and entered it into the statistical analysis as one experimental unit. The field-based blinded counting approach is consistent with published renal immunohistochemical methods [34]. Ten high-power fields provided more than 1000 counted tubular epithelial cells per animal.

### 4.9. Statistical Analysis

The mouse was the experimental unit, and summary data are reported as mean ± SEM. Model residuals were examined for normality with the Shapiro–Wilk test [35] and for equality of variance with Levene’s test [36]. We compared each endpoint across the four groups using one-way ANOVA. When the overall F test was significant, we used Tukey HSD for pairwise contrasts. All analyses were completed in IBM SPSS Statistics 27.0 (IBM Corp., Armonk, NY, USA), and statistical significance was defined as *p* < 0.05.

## 5. Conclusions

Within the defined protocol—20 mg kg^−1^ day^−1^ lycopene for 10 days before a single 200 mg kg^−1^ CP exposure, with evaluation 24 h later—lycopene attenuated selected manifestations of acute renal injury. Relative to CP alone, BUN, pro-inflammatory cytokine transcripts, tubular/tubulointerstitial injury, and cleaved caspase-3 labeling were reduced, whereas serum creatinine, renal TBARS-derived MDA-equivalent values, and SOD activity were not significantly changed. The findings therefore support partial prophylactic renoprotection associated with lower inflammatory transcription and apoptosis/tissue-injury readouts, but they do not demonstrate complete functional recovery or restoration of the measured redox indices. Interpretation is necessarily limited by the single dose and time point, the use of male non-tumor-bearing mice, the absence of cytokine protein and pathway-specific measurements, and the lack of an antitumor-efficacy assessment.

## Figures and Tables

**Table 1:** Primer pairs used to quantify renal transcripts by real-time RT-PCR.

Gene	Forward Primer (5′ → 3′)	Reverse Primer (5′ → 3′)
TNF-α	ATCCATCTCTTTGCGGAGGC	GGGGGAGAGGTAGGGATGTT
IL-1β	TGCCACCTTTTGACAGTGATG	TGATGTGCTGCTGCGAGATT
IL-6	CCCCAATTTCCAATGCTCTCC	CGCACTAGGTTTGCCGAGTA
β-Actin	ACAGTCCATGCCATCACTGCC	GCCTGCTTCACCACCTTCTTG

TNF-α = tumor necrosis factor alpha, IL-1β = interleukin-1 beta, IL-6 = interleukin-6, and β-Actin = beta-actin, encoded by the ACTB gene.

## Data Availability

The original contributions presented in this study are included in the article. Further inquiries can be directed to the corresponding authors.
